# Intranodal implantation of benign thyroid tissue as a late complication of ethanol ablation: A case report

**DOI:** 10.1097/MD.0000000000033381

**Published:** 2022-04-07

**Authors:** Dongbin Ahn, Ji Hye Kawk, Heungrae Cho

**Affiliations:** a Department of Otolaryngology-Head and Neck Surgery, School of Medicine, Kyungpook National University, Daegu, Korea.

**Keywords:** ablation, ethanol, thyroid, thyroid inclusion, tumor seeding

## Abstract

Ethanol ablation (EA) is recommended as the primary treatment for benign thyroid cysts and its use is increasing. Although complications, such as pain, hoarseness, and hematoma, have been reported after EA, implantation of benign thyroid tissue has not been previously reported. Here, we present a case of intranodal implantation of benign thyroid tissue as a late complication of EA.

**Patient concerns:**

A 46-year-old man underwent EA for a benign cystic nodule in the left thyroid lobe and developed a thyroid abscess after several days. The patient was treated with incision and drainage and was discharged without complications. Two years later, the patient presented with multiple masses in both cervical regions. Ultrasound (US) and computed tomography indicated metastatic papillary thyroid carcinoma (PTC) at bilateral levels III, IV, and VI. The results of US-guided fine-needle aspiration cytology (FNAC) indicated benign lesions; however, thyroglobulin levels in the needle washout fluid were >250,000 ng/mL.

**Diagnosis:**

Total thyroidectomy with neck dissection was performed to remove the thyroid and lymph node masses and confirm the diagnosis. Histopathological findings revealed multiple areas of benign thyroid tissue in the bilateral cervical lymph nodes, with no indication of metastatic PTC, even after a BRAF gene mutation study and immunohistochemical staining for HBME-1 and galectin-3.

**Outcomes:**

No recurrence or complications were observed during the follow-up for 29 months.

**Lessons:**

Complicated EA may be associated with the dissemination of benign thyroid tissue into lymph nodes, with a confusing clinical presentation mimicking metastatic PTC. Radiologists and thyroid surgeons should consider the risk of intranodal implantation of benign thyroid tissue as a late complication of EA.

## 1. Introduction

Since the first report of intranodal inclusion of benign thyroid tissue in 1897, its prevalence has ranged from 0.6% to 5.0% of the general population.^[1–4]^ The primary plausible theory for this condition is embryological migration, an entrapment of normal thyroid tissue in the lymph node during embryogenesis.^[1,3,4]^ In fact, most cases with intranodal thyroid inclusion were incidentally identified during pathological examinations after neck dissection for nonthyroidal head and neck cancer, indicating its subclinical characteristics.^[1,2,5]^ However, a recent case report introduced intranodal benign thyroid tissue mimicking nodal metastasis of papillary thyroid carcinoma (PTC) identified on preoperative imaging, in which the diagnosis was eventually confirmed by histopathological examination after total thyroidectomy and neck dissection.^[5]^

Recently, ethanol ablation (EA) and radiofrequency ablation (RFA) have been gaining popularity as nonsurgical and minimally invasive treatments owing to their safety and effectiveness in benign thyroid cysts.^[6–8]^ Accordingly, several international societies and study groups have published guidelines or consensus statements on EA and RFA as the primary treatment modalities for benign thyroid nodules.^[6,7,9]^ However, these techniques are associated with various complications, including pain, hoarseness, and hematoma, which can usually be managed with conservative treatment.^[6,7]^ In rare cases, more severe complications, including nodule rupture or abscess, have been reported, which frequently require surgical intervention.^[9–11]^ However, benign thyroid tissue implantation has not been reported as a complication of EA.

Herein, we report a case of intranodal thyroid tissue implantation following EA for a benign thyroid nodule, introducing the worst consequence of complicated EA and discussing the possible pathogenesis of this phenomenon.

## 2. Case presentation

**Figure 1.**
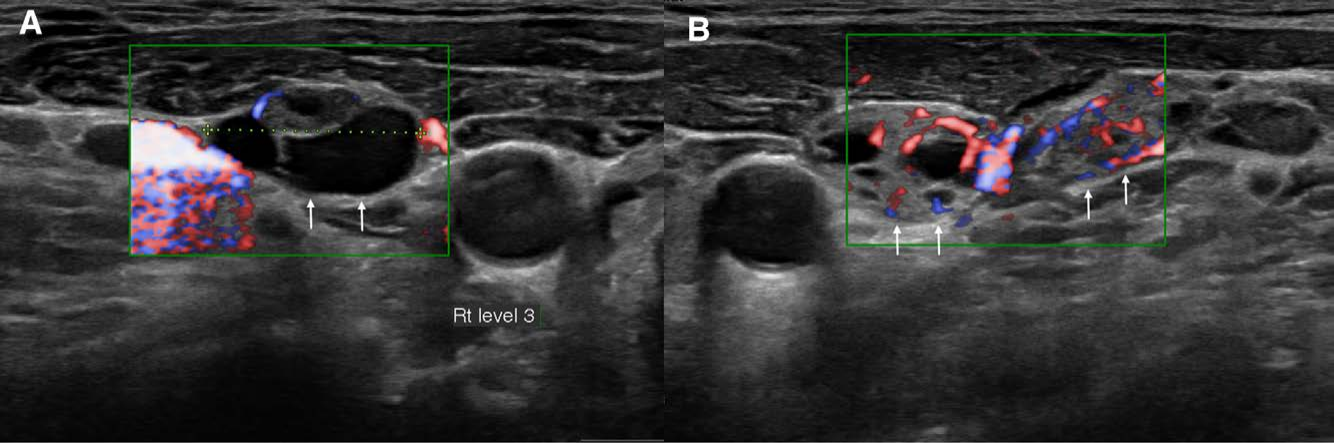
Ultrasonography shows hyperechoic lymph nodes enlargements (arrows) with hypervascularity and cystic changes in the right level III (A) and left level IV (B) regions.

**Figure 2.**
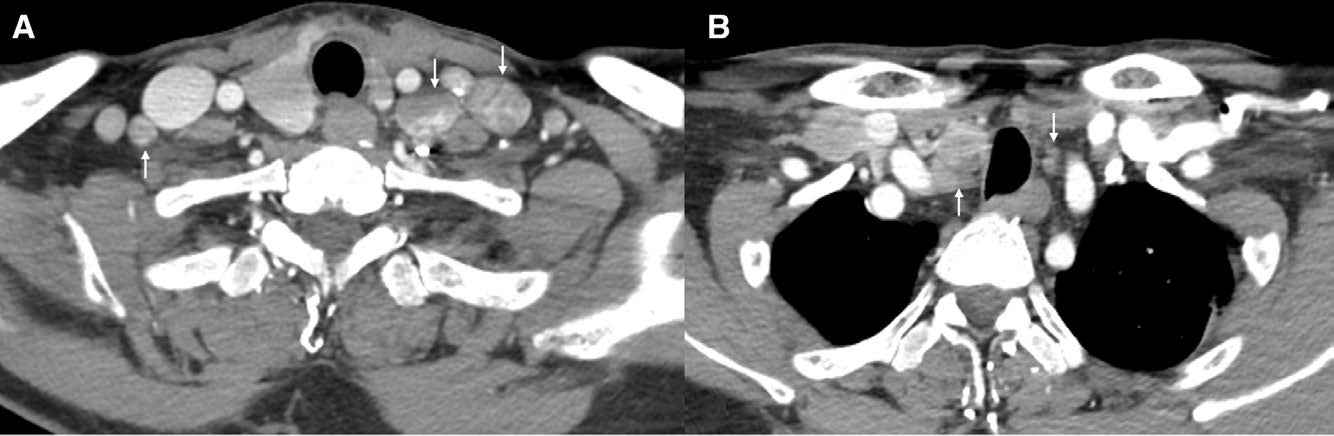
Computed tomography revealed heterogeneously enhanced lymph node enlargements (arrows) in the bilateral level IV (A) and VI (B) regions.

**Figure 3.**
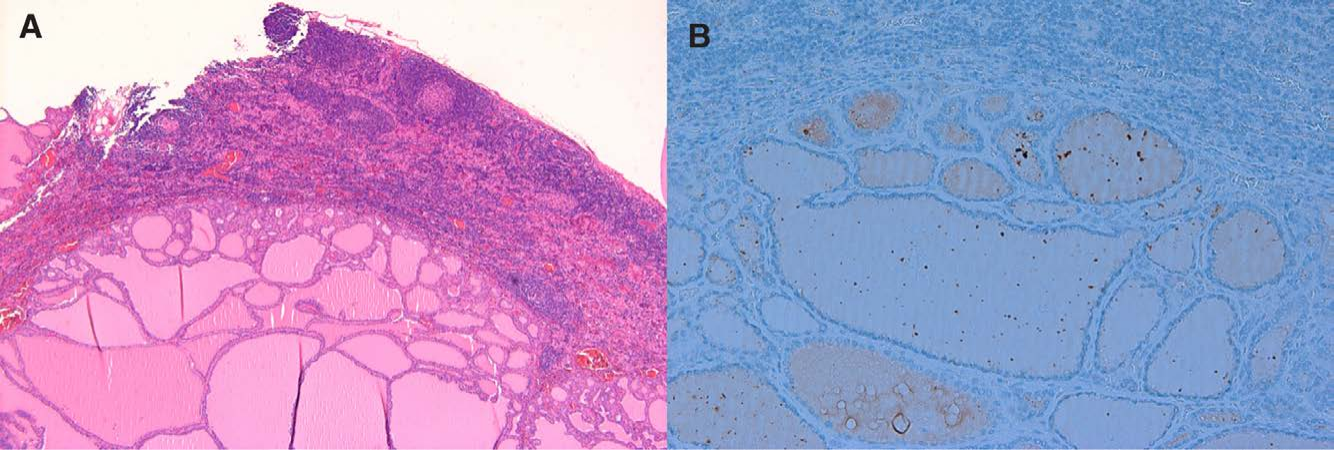
Histopathology of the lymph node shows benign thyroid follicular cells with no atypia (hematoxylin and eosin stain, × 40) (A). HBME-1 immunohistochemical staining is negative in these follicular cells (HBME, × 100) (B).

A 46-year-old man presented a predominantly cystic mass measuring 5.2 × 4.5 × 6.0 cm on the left thyroid lobe. Ultrasound (US)-guided fine-needle aspiration cytology (FNAC) was performed thrice for pathological diagnosis, revealing that the lesion was benign. Therefore, the patient underwent EA. The EA procedure included US-guided aspiration of 15 mL of cystic fluid and subsequent infusion of 10 mL ethanol using an 18-gauge needle. Three days after the procedure, progressive painful swelling developed on the left hemithyroid, which was diagnosed as a thyroid abscess on both US and computed tomography. The patient was treated with incision and drainage and was discharged without complications. Two years later, the patient presented with multiple masses in both the cervical regions. US revealed lymph node enlargement at bilateral levels III, IV, and VI, with heterogeneous hyperechogenicity, hypervascularity, and multifocal cystic changes (Fig. 1). No suspicious nodules were observed in the thyroid gland, although multiple benign cysts were identified in the right hemithyroid and the left lobe was atrophic. Computed tomography confirmed heterogeneously enhanced lymph node enlargement with cystic changes at bilateral levels III, IV, and VI (Fig. 2). These imaging findings were indicative of metastatic PTC. The results of US-FNAC indicated benign lesions; however, thyroglobulin levels in the needle washout fluid were >250,000 ng/mL. Total thyroidectomy and bilateral central and lateral neck dissections were performed to remove thyroid and lymph node masses and confirm the diagnosis. The final histopathological findings revealed benign nodular hyperplasia in both thyroid lobes. In addition, multiple areas of benign thyroid tissue in the bilateral level III, IV, and VI lymph nodes were identified, with no findings indicative of metastatic PTC, even after BRAF gene mutation study and immunohistochemical staining for HBME-1 and galectin-3 (Fig. 3). The patient did not receive adjuvant therapy such as radioactive iodine treatment, and no recurrence was observed during the follow-up period of 29 months.

## 3. Discussion

To our knowledge, this is the first report of intranodal implantation of benign thyroid tissue after EA. In addition to its uniqueness, this case is clinically significant as it suggests that EA can be associated with the dissemination of benign thyroid tissue into the lymph nodes, which may lead to a confusing clinical presentation mimicking metastatic PTC.

Although EA and RFA are less invasive and safer techniques than surgical treatment of thyroid nodules, both procedures inevitably entail capsule disruption of the nodule. Therefore, these techniques potentially pose the risk of tumor seeding associated with needle puncture. Moreover, the increased risk of tumor seeding is generally associated with the use of larger needles.^[12]^ Although there is a lack of studies reporting tumor seeding after EA for benign thyroid nodules, the risk can be assumed using data from core-needle biopsy (CNB) as the needle bore used for EA (16–20 gauge) is similar to that used in CNB.^[7]^ In a systematic review of tumor seeding after FNAC and CNB for head and neck tumors, the crude incidence of tumor seeding after CNB was 0.0011%. With respect to tumor seeding after ablation of the thyroid gland, only 2 cases of needle track seeding have been reported after RFA, and the thyroid tumors in both cases were finally diagnosed as thyroid cancer.^[13,14]^

Interestingly, our case was quite different from cases of tumor seeding after needle biopsy or tumor ablation, as thyroid tissue was not found along the needle pass track, but had been disseminated to the bilateral level III, IV, and VI lymph nodes, consistent with the typical drainage path of thyroid lymphatics. Furthermore, the histopathological results demonstrated that the disseminated thyroid tissue was benign, in contrast to the findings reported in the previous 2 cases of thyroid cancer seeding after RFA.^[13,14]^ Therefore, it is strongly suggested that intranodal implantation of benign thyroid tissue in this case developed by transport of thyroid tissue through the thyroid lymphatic systems rather than simple tumor displacement resulting from capsule disruption. The pathogenesis of this phenomenon is associated with thyroid tissue destruction by injecting ethanol, with or without subsequent infection, which disintegrates the thyroid lymphatic system, allowing the incorporation of separated thyroid tissue into the lymphatic system. In fact, the pathogenesis of benign thyroid inclusions in the cervical lymph nodes has been a subject of debate in many previous studies.^[1–4]^ Although the most plausible hypothesis is the embryological migration theory, which involves entrapment of thyroid tissue within the lymph node during embryogenesis. This case shows that lymphatic transport can be the primary mechanism of intranodal thyroid inclusions under specific clinical conditions, particularly in patients undergoing ablation treatment.^[1,3,4,15]^

Benign thyroid tissue implantation after EA is extremely rare, and uncomplicated EA is unlikely to be associated with this phenomenon. Despite its rarity, physicians should consider the possibility of this condition in patients undergoing EA or RFA, as intranodal thyroid tissue implantation highly mimics metastatic thyroid carcinoma, leading to unnecessary radical surgical treatment. As shown in this case, it is difficult to differentiate intranodal benign thyroid tissue from metastatic PTC based on imaging studies alone. Therefore, physicians should have a high clinical suspicion if findings suggestive of metastatic PTC lymph nodes are identified in patients with a history of previous ablation treatment for benign thyroid nodules with no current suspicion of thyroid nodules, provided the lymph node FNAC indicates benign findings and thyroglobulin levels are high in needle washout fluid. In such cases, it would be reasonable to perform additional CNB or excisional biopsy to confirm the preoperative diagnosis of metastatic PTC or intranodal benign thyroid tissue rather than performing immediate therapeutic surgery to avoid unnecessary radical surgery.

In conclusion, this case shows that complicated EA can result in intranodal implantation of benign thyroid tissue, mimicking metastatic PTC, even several years after EA. Although this is an extremely rare complication, it may become more common in the future, as the use of EA has been increasing since its recommendation as the primary treatment for benign thyroid cysts. Therefore, radiologists and thyroid surgeons should consider the risk of intranodal implantation of benign thyroid tissue as a late complication of EA, and long-term follow-up should be performed for patients undergoing EA.

## Author contributions

**Conceptualization:** Dongbin Ahn, Ji Hye Kawk.

**Data curation:** Dongbin Ahn, Ji Hye Kawk.

**Investigation:** Dongbin Ahn, Heungrae Cho.

**Supervision:** Dongbin Ahn.

**Validation:** Heungrae Cho.

**Writing – original draft:** Dongbin Ahn, Ji Hye Kawk.
